# Carboxymethyl chitosan hydrogel formulations enhance the healing process in experimental partial-thickness (second-degree) burn wound healing

**DOI:** 10.1590/ACB360303

**Published:** 2021-04-05

**Authors:** Randys Caldeira Gonçalves, Roberta Signini, Luciana Martins Rosa, Yuri Santana Pereira Dias, Marina Clare Vinaud, Ruy de Souza Lino

**Affiliations:** 1 PhD. Universidade Federal de Goiás – Instituto de Patologia Tropical e Saúde Pública – Programa de Pós-Graduação em Medicina Tropical e Saúde Pública – Goiânia (GO), Brazil.; 2 PhD. Universidade Estadual de Goiás – Campus de Ciências Exatas e Tecnológicas – Anápolis (GO), Brazil.; 3 Graduate student. Universidade Federal de Goiás – Faculdade de Medicina – Goiânia (GO), Brazil.; 4 PhD. Universidade Federal de Goiás – Instituto de Patologia Tropical e Saúde Pública – Departamento de Biociências e Tecnologia – Goiânia (GO), Brazil.

**Keywords:** Hydrogels, Carboxymethyl Chitosan, Experimental Burn Wound, Rats, General Pathologic Processes

## Abstract

**Purpose:**

This study aimed to elaborate a hydrogel constituted by carboxymethyl chitosan (CMC), hyaluronic acid (HA) and silver (Ag) and to evaluate its healing effect on partial-thickness burn wounds experimentally induced in rats.

**Methods:**

CMC was obtained by chitosan reacting with monochloroacetic acid. The carboxymethylation was confirmed by Fourier-transform infrared spectroscopy and hydrogen nuclear magnetic resonance (NMR). Scanning electron microscopy was used to determine the morphologicalcharacteristics of chitosan and CMC. After the experimental burn wound induction, the animals (n = 126) were treated with different CMC formulations, had their occlusive dressings changed daily and were followed through 7, 14 and 30 days. Morphometric, macroscopic and microscopic aspects and collagen quantification were evaluated.

**Results:**

Significative wound contraction, granulation tissue formation, inflammatory infiltration and collagen fibers deposit throughout different phases of the healing process were observed in the CMC hydrogels treated groups.

**Conclusions:**

The results showed that, in the initial phase of the healing process, the most adequate product was the CMC/HA/Ag association, while in the other phases the CMC/HA association was the best one to promote the healing of burn wounds.

## Introduction

Burn wounds are tissue injuries that may present partial or total destruction of the external covering tissue of the body. It may reach deep tissues, such as subcutaneous, muscle, tendons and even bones^1^. The main causes of burn wounds are heat, electricity, radiation, friction or contact with chemical agents. Its severity is directly related to the depth of the injury and it is classified as superficial-thickness burn wound (first degree), partial-thickness burn wound (second degree) and full-thickness burn wound (third degree)^1^,^2^.

Such injuries are considered a severe public health issue^1^,^3^ and the World Health Organization (WHO) estimates that about 24 million people suffer burn wounds each year around the world, most of them in low- and medium-income countries^4^. In Brazil, it is estimated that about a million people are victims of some kind of thermic trauma each year and 2,500 die directly or indirectly due to these injuries^5^.

An ideal dressing for a burn wound should act directly on the site of the injury, stimulating the healing process. The dressings should have biological properties in order to ensure a high humidity environment, absorb exudates and toxic components from the wound, allow gas exchange and avoid bacterial contamination^6^. Besides, the dressing should not be toxic nor allergenic and should be composed by biocompatible materials^7^,^8^.

Hydrogels are innovative biomaterials used in injuries dressings. Their tridimensional structure acts as a barrier against microorganisms, facilitates adhesion and cell proliferation, and its permeability enables the gas exchange necessary for tissue respiration^9^,^10^. Furthermore, hydrogels are easily rinsed, they remove the exudate and create a humid environment on the injury interface^11^–^14^.

There are several types of dressings used in the burn wounds treatment, such as polymeric hydrogels and silver (Ag) incorporated dressings. Hydrogels may present different formulations, for instance, using chitosan (linear polymer of N-acetyl-D-glucosamine and a deacetylated glucosamine); diverse materials, which result in structural modifications; and hyaluronic acid ([HA] linear glycosaminoglycan composed by units of glucuronic acid and n-acetylglucosamine). The use of hydrogels as dressings in burn wounds has been recommended due to their essential characteristic of biological dressings, such as hydrophilicity, biodegradability, nonimmunogenic nature and healing properties^15^–^17^.

Carboxymethyl chitosan (CMC) is a carboxymethylated derivative of the chitosan polymer, which presents higher biocompatibility and solubility in water than chitosan^18^, and better antimicrobial performance^19^. While the HA presents several biological activities, such as elasticity of the intercellular tissue, fibrogenesis, angiogenic effect, anti-inflammatory and immunosuppressive characteristics^20^. Silver dressings are widely used in the treatment of burn wounds because they improve re-epithelialization, reduce levels of pain and decrease bacterial infections^21^. In spite of the use of nanoparticles and Ag salts being controversial due to its apparent toxicity, it has been shown that, when the polymeric hydrogels are associated to nanoparticles and Ag salts, the cutaneous toxicity is absent as there is a gradual liberation within the tissue^7^,^22^.

The association of Ag and chitosan hydrogel induces a significant contraction of the wound, resulting in a quicker healing process. Also, the association of Ag with HA aids in the repair and regeneration of the injury^15^,^23^–^25^. On the other hand, the combination of chitosan and HA induced greater re-epithelialization of the wound^14^,^26^,^27^. Therefore, it is believed that the production of hydrogels of CMC incorporated with HA and Ag would result in a promising dressing with bioactive properties and increased efficacy regarding the healing of the wound.

This study aimed to evaluate the healing process of experimentally induced burn wounds in rats treated with CMC hydrogel incorporated with HA and Ag.

## Methods

### Carboxymethyl chitosan synthesis

Carboxymethyl chitosan was synthesized via heterogeneous carboxymethylation reaction based on the method described by Liu *et al*.^28^. Briefly, 10 g of chitosan in 220 mL of isopropanol were added under mechanical stirring (600 rpm) for 15 min. Soon after, 68 g of sodium hydroxide solution (50%) were added. This mixture was stirred for 1 h at room temperature. A solution of monochloroacetic acid (15 g) in isopropanol (20 mL) was then dripped onto the previously prepared mixture and the reaction was maintained for 24 h under constant stirring and at room temperature.

The mixture formed was filtered, washed with 70% ethanol. The synthesized CMC was suspended in 500 mL of methanol (80%) and left under constant stirring for 30 min. The suspension was neutralized with glacial acetic acid until neutral pH. Soon after, the suspension was filtered again and the obtained product was washed with absolute ethanol and dried at room temperature until constant weight.

### Hydrogen nuclear magnetic resonance analysis(^1^H NMR)

High resolution hydrogen NMR spectroscopy was used to characterize the original chitosan and the synthesized CMC. The spectrum was obtained at the NMR laboratory of the Chemistry Institute of the Universidade Federal de Goiás. Approximately 10 mg of the sample (chitosan and CMC) were added to 1.0 mL of D_2_O/HCl mixture(1% v/v) and left under constant stirring for 24 h, resulting in a clear, viscous solution.

The ^1^H NMR spectra were acquired on a Bruker spectrometer operating at 500 MHz at 80 °C. The interval between pulses was 3 seconds, 32 scans and the relaxation time was 7 seconds. Chemical shifts (δ) were expressed in dimensionless values (ppm) in relation to an internal tetramethylsilane (TMS) standard^29^. The visualization of the spectra was performed using the ACD LABS 12.0 software. The determination of the average degree of substitution (% GS) was carried out using the following Eqs. 1–4 30:

$$f_{6}=\left(\frac{1}{2}\right)\frac{A_{a}-A_{b}}{A_{H_{2}}}$$ (1)

$$f_{3}=\frac{A_{b}}{A_{H_{2}}}$$ (2)

$$f_{2}=\left(\frac{1}{2}\right)\frac{A_{c}}{A_{H_{2}}}$$ (3)

$$F=f_{6}+f_{3}+f_{2}$$ (4)

where: *A_a_* = area of the two hydrogens of the carboxymethyl group linked to C-6 and one hydrogen of the carboxymethyl grouplinked to C-3; *A_b_* = area of a hydrogen of the carboxymethyl group linked to C-3; *A_c_* = area of the two hydrogens of the carboxymethyl group linked to N linked to C-2; and *A_H2_* = area of hydrogen bonded to C-2 carbon.

The values obtained in *f*_6_, *f*_3_ and *f*_2_ correspond to the carboxymethylation fractions at positions 6-O-, 3-O- and 2-N-, respectively, and F is the total fraction of carboxymethylation^30^.

### Infrared spectroscopy analysis

Infrared analyses were performed on KBr tablets using the spectrum Frontier full time infrared / near infrared (FTIR/NIR) spectrometer PerkinElmer (Perkin-Elmer Corp., Norwalk, CT) (Laboratório de Análise Instrumental do Campus de Anápolis de Ciências Exatas e Tecnológicas Henrique Santillo da Universidade Estadual de Goiás [CCET-UEG]), in order to observe the absorption bands characteristic of the original chitosan and the synthesized CMC. The FTIR spectral analysis^31^ was performed within the range of wave numbers from 400 to 4000 cm^–1^, with a resolution of 4 cm^–1^.

### Hydrogel formulations

Four different hydrogel formulations were prepared: (i) CMC, (ii) CMC and HA, (iii) CMC and Ag, (iv) CMC, HA and Ag). The hydrogels presented the following constant concentrations: CMC 2%, HA 0.2%, Ag nitrate 1% in sterile deionized water. The hydrogels were prepared inside a flow chamber in aseptic conditions in 1.5 mL sterile centrifuge tubes (Eppendorf), and were stored at –8 °C. The hydrogels formation was confirmed visually through the inverted flask method^25^.

### Scanning electron microscopy (SEM) analysis

A Jeol scanning electron microscope, JSM-6610, equipped with EDS, Thermo scientific NSS Spectral Imaging (Laboratório Multiusuário de Microscopia de Alta Resolução, Instituto de Física, UFG) was used to observe the morphology of the original chitosan, of the CMC synthesized and lyophilized hydrogel. The samples were fixed on an aluminum plate with the aid of double-sided graphite adhesive tape, covered by a thin layer of gold approximately 20 nm thick.

### Thermogravimetric analysis

The thermal profiles of samples of the synthesized CMC and the hydrogels previously lyophilized were performed in the instrumental analysis laboratory of CCET/UEG, Anápolis, GO, Brazil. Approximately 30 mg of each sample was submitted to a temperature range of 25 to 800 °C, using a heating rate of 10 °C·min^–1^, under a dynamic nitrogen atmosphere, with gas flow in the order of 20 mL·min^–1^.

### Burn wound healing studies

This study was approved by the Ethics Committee in Animal Use of the Universidade Federal de Goi**á**s, protocol number 118/17.

A total of 126 Wistar (*Rattus norvegicus albinus*) rats 60 days old with a medium weight of 150 to 200 g were used. The animals were maintained in cages with free access to water and ration, with controlled temperature (24 ± 1 °C) with a light/dark cycle of 12 h. The animals were divided into 7 subgroups containing 18 animals each, according to the treatment received, as follows: 1) PS – physiologic solution (NaCl 0.9%); 2) SS – 1% silver sulfadiazine (Prati-Donaduzzi, Brazil, batch 16A273), 3) Hy – 0.2% Hyaludermin (Systagenix, United Kingdom, batch 16804) commercial HA ointment; 4) CMC hydrogel; 5) CMC/HA hydrogel, 6) CMC/Ag hydrogel, 7) CMC/HA/Ag hydrogel. Groups 1, 2 and 3 were considered control groups, while groups 4 to 7 were the ones treated with hydrogel formulations and, therefore, considered the test groups. The animals were followed through 3 experimental days, 7, 14 and 30 days. At each experimental day, six animals from each group were euthanized.

### Burn wound induction, dressings and euthanasia

On day zero, the animals were weighted, anesthetized through intraperitoneal administration of 0.1mL/100 gof animal weigh of ketamine 10% (União Química Farmacêutica Nacional S/A, Brazil), xylazine 2% (Sespo Indústria e Comércio Ltda, Brazil) and injection water. After the anesthesia, the dorsal region of the animal was prepared with trichotomy and antisepsis with 70% alcohol. The burn wound injuries were induced by scalding at 96 ℃. The animals were gently maneuvered inside a polyvinyl chloride cylindric tube with a 2 × 2 cm^2^ opening and sealed extremities^32^–^34^.

The animals received daily occlusive sterile dressings hydrated with physiologic solution. The injuries were protected with physiologic solution humidified sterile gauze and cotton cloth. The specific topic agent of each treatment group was applied in a uniform layer sufficient to cover all the wound bed. The crusts on the wound bed were removed before the appliance of the dressings using tweezers and humidified gauze in order to prevent difficulty in the appliance of the product and its contact to the injury bed.

After the induction of the burn wound three animals were maintained per cage until the euthanasia day 7, 14 and 30, which was performed using a CO_2_ chamber. The animals submitted to the burn wound induction were followed in order to detect pain and/or suffering by a veterinarian physician and received analgesic treatment during the first seven days with tramadol hydrochloride (Grünenthal Brasil Farmacêutica Ltda) diluted in the drinking water and with a daily intraperitoneal injection of tramadol, dosage of 12.5 mg/kg of body weight.

### Morphometric evaluation of the wound contraction

The dimensions of the wound were measured with a ruler and photographed at day zero (burn wound induction day) and at days 14 and 30. The following formula was used to calculate the percentage of wound contraction (Eq. 5):

$$\mathrm{CD}=\left[\frac{\left(\text{Area}D^{0}-\text{Area}D^{\text{Euthanasia}}\right)}{\text{Area}D^{0}}\right]\times 100$$ (5)

CD = contraction degree; D0 = day of burn wound induction; D Euthanasia = Days 14 and 30 after the injury induction.

The delimitation of the burn wound area was performed through the Image J software.

### Macroscopic evaluation

At experimental days 7, 14 and 30 after the burn wound induction, the phases of the healing process (inflammatory, proliferative and remodeling) were macroscopically analyzed. The following parameters were evaluated: necrosis/crust, granulation tissue and re-epithelialization. The analysis of the pathologic processes was qualitative, as follows: absent (score 0), discrete (up to 25% of tissue commitment, score 1); moderate (between 26 and 50% of tissue commitment, score 2) and accentuated (more than 50% of tissue commitment, score 3)^32^–^34^.

### Microscopic evaluation

The microscopic evaluation was performed in fragments of the wound removed after the euthanasia and fixed in 10% buffered formaldehyde (pH 7.2) for 48 h, posteriorly included in paraffin, submitted to 4 μ transversal cuts using a microtome (Leica RM2255). The slides were stained by hematoxylin and eosin (H&E) and by picrosiriusred (PS).

In the H&E-stained slides, the pathologic processes evaluated were polymorphonuclear cells inflammatory infiltration, mononuclear cells inflammatory infiltration and angiogenesis throughout the whole extension of the slide. These parameters were identified in a semi-quantitative form, as follows: absent (score 0), discrete (up to 25% of slide commitment, score 1), moderate (from 26 to 50% of slide commitment, score 2), accentuated (more than 50% of slide commitment, score 3).

The collagen quantification was performed in the PS-stained slides under polarized light. The analysis was performed using the Image J software (National Institutes of Health, EUA).

### Statistical analysis

All statistical analysis was performed using the Sigma Stat 2.3 software. All variables were tested in order to determine the normal distribution. The ones which presented normal distribution were analyzed through parametric tests, while the non-normal distribution variables were analyzed through nonparametric tests. In the morphometric analysis, according to the normal distribution and homogeneous variance, the analysis of variance (ANOVA) parametric test, followed by Tukey’s post-test were used. In the macroscopic and microscopic analysis, according to the non-normal distribution, the nonparametric test Kruskal-Wallis followed by Dunn’s post-test. All data are presented as mean and standard deviation or as median with maximum and minimum values. All analysis were considered significant when p ≤ 0.05.

## Results

### Hydrogen nuclear magnetic resonance spectroscopy and infrared spectroscopy

**Figure 1:**
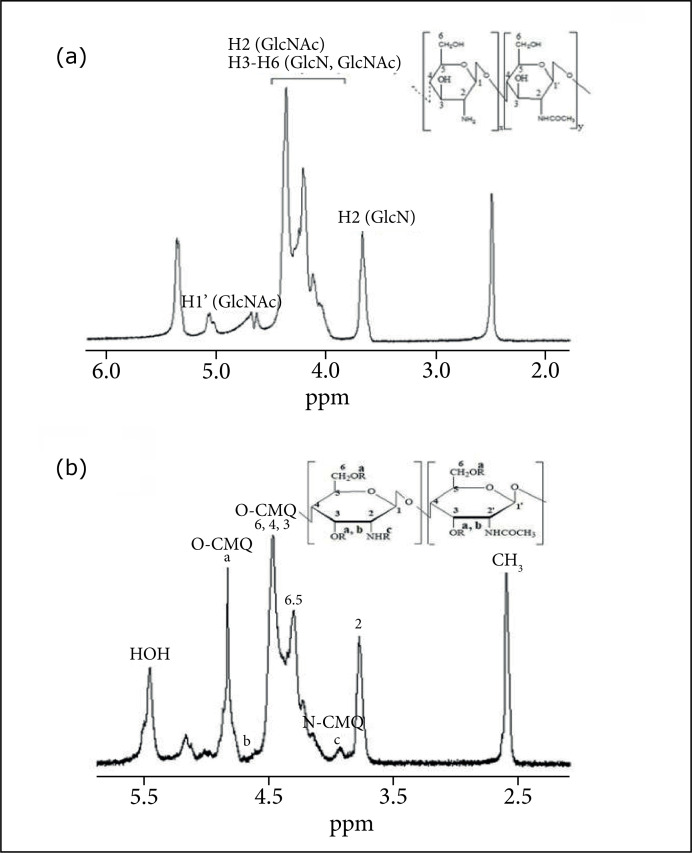
Hydrogen nuclear magnetic resonance spectrum of (a) chitosan and (b) CMC. GlcNAc = N-acetylglucosamine and GlcN = glucosamine. The ^1^H NMR spectra were determined using Bruker operating at 500 MHz at 80 °C,with a 3 s interval between pulses, 32 scans and a 7 s relaxation time. The visualization of the spectra was performed using the ACD LABS 12.0 software.

The comparison of the chitosan spectra (Fig. 1a) with the CMC (Fig. 1b) suggests that in the carboxymethylation reaction the carboxymethyl groups (carboxylic acid) were effectively introduced into the polymeric matrix, proving the success of the synthesis. The ^1^H NMR spectrum of chitosan exhibited a signal around 3.65 ppm related to the C2-linked hydrogen atom of the glucosamine ring (GlcN), a 2.5 ppm singlet characteristic of the methyl hydrogens of GlcNAc units (acetamide). The signals at 4.0 and 4.5 ppmare attributed to the hydrogen H3-H6 present in the GlcNA ring (glycopyranoside), while the signals occurring at 5.07 ppmare attributed to hydrogen bound to the anomeric carbon (C1) of the N-acetylglycosamine ring (H1) and the peak at 5.35 ppm is due to hydrogen bound to the anomeric carbon (C1) of the glycosamine ring (H1).

Figure 1b represents the ^1^H NMR spectra of CMC, in which “a” corresponds to the two hydrogens of the carboxymethyl group attached to C (6) and the hydrogen of the carboxymethyl group attached to C (3) (–CH2COOD 4.6 < δ < 4.9 ppm), band “b” corresponds to the hydrogen of the carboxymethyl group linked to C (3) (–CH2COOD 4.5 < δ < 4.6 ppm). The “c” band corresponds to the two hydrogens of the carboxymethyl group linked to the nitrogen of the amino group (- + ND2CH2COOD 3.8 < δ < 4.0 ppm), showing N-carboxymethylation. However, N-carboxymethylation was very low, showing that the carboxymethylation occurred is predominant in the hydroxyl group.

From the analysis of the NMR-1 of the CMC and the Lamas equations (2008), the value obtained from the average degree of substitution in the carboxymethylation reaction, that is the number of carboxymethyl groups (-CH2COOH) inserted in the polymer chain per monomer unit determined, was 1.42.

**Figure 2:**
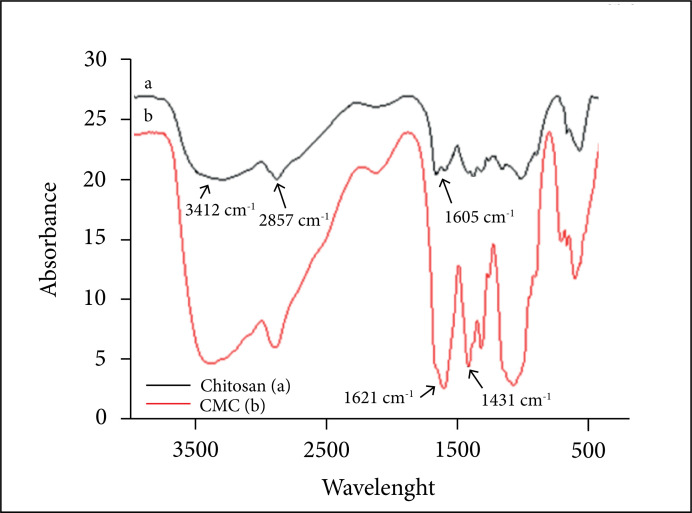
Fourier-transform infrared spectroscopy of (**a**) chitosan (black line) and (**b**) CMC (red line).

The FTIR spectra of chitosan (Fig. 2a) showed an absorption band with a peak at 3412 cm^–1^ attributed to the stretching vibrations of the OH and NH bonds in the molecule. The absorption of the band at 1662 cm^–1^ is attributed to the elongation of C = O of amides. The 1605 cm^–1^ band results from the angular deformation of the NH bonds of the amino groups. There are also absorptions between 1643 and 2857 cm^–1^, respectively, characteristics of the angular strain of the NH bonds of the amino groups and symmetric axial strain of the CH and CH2 groups. For CMC (Fig. 2b), an intense band at 1621 cm^–1^ and a moderate band at 1431 cm^–1^ were identified, due to the symmetry of −COOH asymmetric axial deformations, respectively, which confirms the introduction of the carboxymethyl groups.

### Morphology of the lyophilized hydrogels

**Figure 3:**
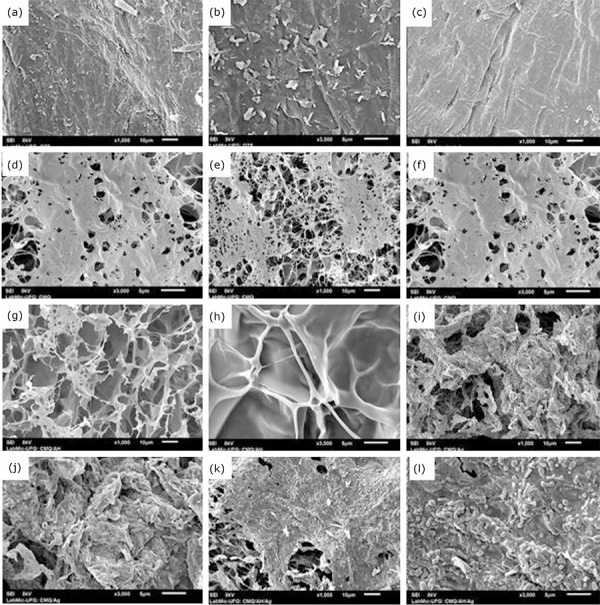
Scan electron micrographs of the surfaces of the (**a**) original chitosan (scale 10 μm); (**b**) original chitosan (scale 5 μm); (**c**) synthetized CMC (scale 10 μm); (**d**) synthetized CMC (scale 5 μm); (**e**) CMC lyophilized hydrogel (scale 10 μm); (**f**) CMC lyophilized hydrogel (scale 5 μm); (**g**) CMC/HA lyophilized hydrogel (scale 10 μm); (**h**) CMC/HA lyophilized hydrogel (scale 5 μm); (**i**) CMC/Ag lyophilized hydrogel (scale 10 μm); (**j**) CMC/Ag lyophilized hydrogel (scale 10 μm); (**k**) CMC/HA/Ag lyophilized hydrogel (scale 10 μm); (**l**) CMC/HA/Ag lyophilized hydrogel (scale 5 μm).

Chitosan and CMC had a continuous, irregular and rough surface (Fig. 3a–d). The surface of the lyophilized hydrogel of CMC (Fig. 3e and f) and CMC/HA (Fig. 3g and h)showed well-developed porosities and the hydrogel of CMC/Ag (Fig. 3i and j) and CMC/HA/Ag (Fig. 3k and l)had a surface with reliefs and a fibrous appearance under the polymeric matrix.

### Thermogravimetric analysis (TGA)

**Figure 4:**
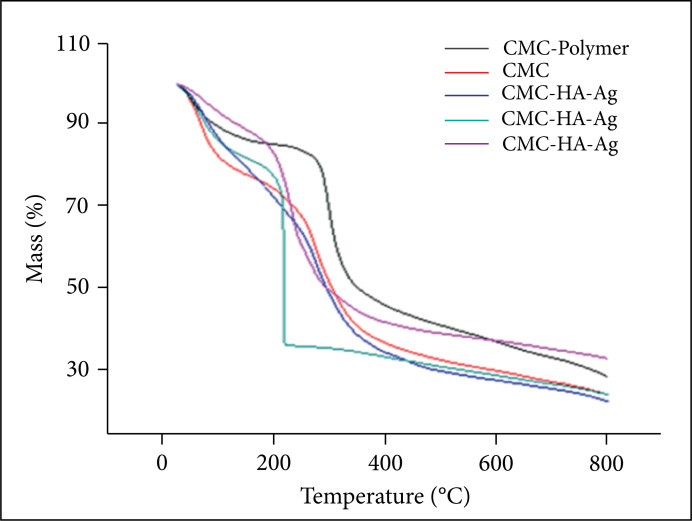
Thermogravimetry curve of CMC and lyophilized hydrogels. CMC: carboxymethyl chitosan hydrogel; CMC/HA: carboxymethyl chitosan hydrogel and hyaluronic acid; CMC/Ag: carboxymethyl chitosan and silver hydrogel; CMC/HA/Ag: hydrogel of carboxymethyl chitosan, hyaluronic acid and silver.

The TGA analysis showed three processes of mass loss, the first being related to dehydration (40 to 150 °C),followed by thermal degradation of the pyranose ring of the polymer chain (290 to 310 °C) and the decomposition of the carboxylic groups and glucosamine units of the polysaccharide structure (above 500 °C) (Fig. 4).

### Morphometric analysis

In the inflammatory phase, the groups treated with CMC, CMC/HA, CMC/Ag presented greater wound contraction than all the control groups. When comparing the hydrogel treatments, the CMC/HA/Ag presented lower wound contraction than the other treatments.

While in the proliferative phase, the groups treated with CMC, CMC/HA, CMC/Ag presented greater wound contraction than the PS control group. Regarding the hydrogel treatments, the CMC/HA/Ag presented lower wound contraction than the other treatments.

**Figure 5:**
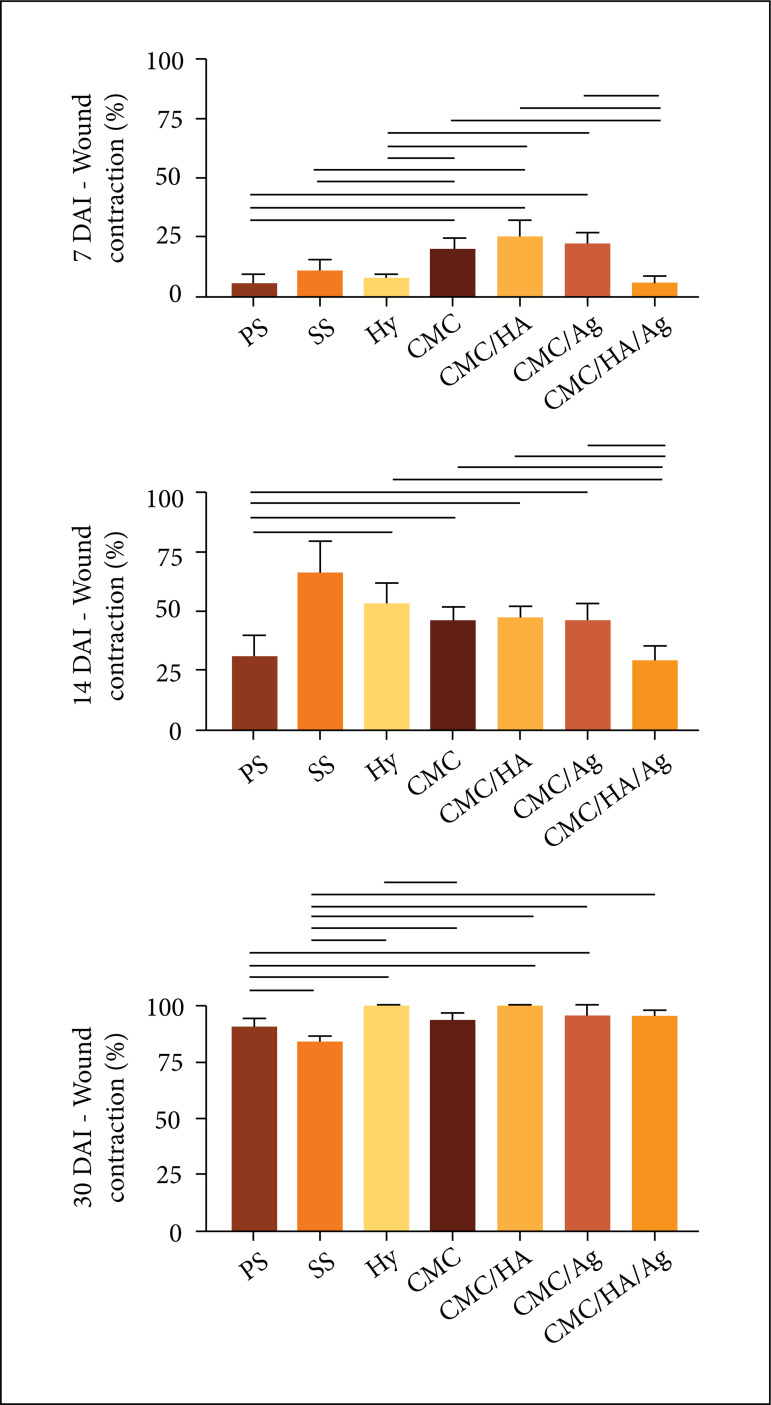
Morphometric analysis of the contraction degree of partial-thickness burn wounds experimentally induced in Wistar rats. Results expressed in mean ± standard deviation of the percentage of contraction. n: number of animals. DAI: days after the injury induction. PS – physiologic solution (NaCl 0.9%); SS – 1% silver sulfadiazine; Hy – 0.2% Hyaludermin; CMC – carboxymethyl chitosan; CMC/HA – carboxymethyl chitosan and hyaluronic acid; CMC/Ag – carboxymethyl chitosan and silver; CMC/HA/Ag – carboxymethyl chitosan, hyaluronic acid and silver. Horizontal lines indicate the significant statistical difference. Statistical analysis: ANOVA and Tukey’s post-test.

On the other hand, during the remodeling phase only the groups treated with CMC/HA and CMC/Ag presented greater wound contraction than the PS and SS control groups. Comparing the hydrogel treatments, the CMC treated group presented lower wound contraction than the CMC/HA treated one (Fig. 5).

### Macroscopic analysis

The macroscopic aspects of the healing process of partial-thickness burn wounds experimentally induced in rats were compared regarding necrosis/crust, granulation tissue and re-epithelialization in the inflammatory (between 1^st^ and 7^th^ days), proliferative (between 7^th^ and 14^th^ days) and remodeling (between 14^th^ and 30^th^ days) phases of the healing process (Table 1).

**Table 1:** Macroscopic analysis of pathological processes in partial-thickness burn wounds experimentally induced in Wistar rats.

	DAI	Control (n = 18)			Hydrogels (n = 18)				p	Dunn’s
		PS	SS	Hy	CMC	CMC/HA	CMC/Ag	CMC/HA/Ag		
Necrosis/crust	7	3 (2–3)	0 (0–1)	3 (3–3)	3 (2–3)	3 (2–3)	2.5 (2–3)	3 (3–3)	< 0.001*	Hy > SS CMC/HA/Ag > SS PS > SS CMC/HA > SS CMC > SS CMC/Ag > SS
	14	2 (1–3)	1 (0–2)	0 (0–2)	0.5 (0–1)	0.5 (0–1)	1.5 (0–3)	0 (0–1)	0.027*	PS > CMC/HA/Ag PS > CMC/HA PS > CMC PS > Hy
	30	2 (2–3)	0 (0–1)	0 (0–0)	0 (0–1)	0 (0–0)	0 (0–1)	0 (0–0)	< 0.001*	PS > CMC/HA/Ag PS > CMC/HA PS > Hy PS > CMC/Ag PS > SS PS > CMC
Granulation tissue	7	2 (1–2)	0 (10–0)	0 (0–0)	0 (0–1)	0 (0–1)	1 (0–2)	0 (0–1)	< 0.001*	PS > Hy PS > SS PS > CMC/HA/Ag PS > CMC/HA PS > CMC CMC/Ag > Hy CMC/Ag > SS CMC/Ag > CMC/HA/Ag CMC/Ag > CMC/HA CMC/Ag > CMC
	14	2.5 (2–3)	1 (0–2)	3 (3–3)	3 (3–3)	3 (3–3)	2 (1–3)	3 (2–3)	> 0.001*	Hy > SS CMC > SS CMC/HA > SS CMC/HA/Ag > SS PS > SS
	30	0 (0–0)	1 (0–1)	0 (0–0)	0 (0–0)	0 (0–0)	0 (0–0)	0 (0–0)	> 0.001*	SS > CMC/HA/Ag SS > CMC/HA SS > CMC/Ag SS > CMC SS > Hy SS > PS
Re-epithelialization	7	0 (0–0)	0 (0–0)	0 (0–0)	0 (0–0)	0 (0–0)	0 (0–0)	0 (0–0)	1	
	14	0.5 (0–2)	0 (0–1)	1 (1–1)	1 (0–1)	1 (0–1)	0 (0–1)	1 (0–1)	0.126	
	30	3 (2–3)	3 (3–3)	3 (3–3)	3 (3–3)	3 (3–3)	3 (3–3)	3 (3–3)	0.022*	CMC > PS CMC/HA > PS CMC/Ag > PS CMC/HA/Ag > PS SS > PS Hy > PS

Results expressed in median (minimum – maximum). n: number of animals. DAI: days after the injury induction. PS: physiologic solution (NaCl 0.9%); SS: 1% silver sulfadiazine; Hy: 0.2% Hyaludermin; CMC: carboxymethyl chitosan; CMC/HA: carboxymethyl chitosan and hyaluronic acid; CMC/Ag: carboxymethyl chitosan and silver; CMC/HA/Ag: carboxymethyl chitosan, hyaluronic acid and silver. Statistical analysis: Kruskal Wallis and Dunn’s post-test.

* p < 0.05.

In the inflammatory phase, the groups treated with CMC, CMC/HA and CMC/HA/Ag presented lower granulation tissue than the PS control group. Regarding the comparison between treatments, the CMC, CMC/HA and CMC/HA/Ag treated groups presented lower granulation tissue than the CMC/Ag treated one.

In the proliferative phase, the groups treated with CMC, CMC/HA and CMC/HA/Ag presented lower necrosis/crust than the PS control group.

While, in the remodeling phase, the groups treated with all the hydrogel formulations presented lower necrosis and greater re-epithelialization than the PS control group.

### Microscopic analyses

**Figure 6:**
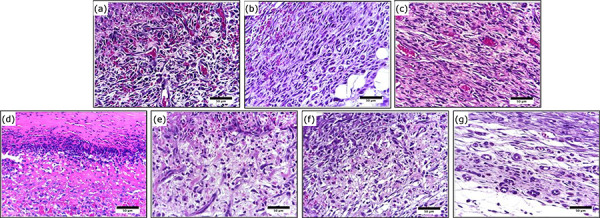
Photomicrograph of partial-thickness burn wounds experimentally induced in Wistar rats 7 days after the injury induction. (**a**) Physiologic solution (NaCl 0.9%) treatment, (**b**) 1% SS treatment, (**c**) 0.2% Hy, (**d**) CMC hydrogel treatment, (**e**) CMC/HA hydrogel treatment, (**f**) CMC/Ag hydrogel treatment, (**g**) CMC/HA/Ag hydrogel treatment. Objective × 40. Scale in μm.

**Figure 7:**
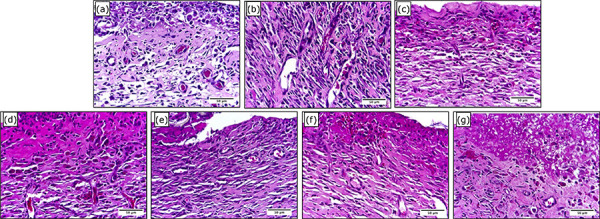
Photomicrograph of partial-thickness burn wounds experimentally induced in Wistar rats 14 days after the injury induction. (**a**) Physiologic solution (NaCl 0.9%) treatment, (**b**) 1% SS treatment, (**c**) 0.2% Hy, (**d**) CMC hydrogel treatment, (**e**) CMC/HA hydrogel treatment, (**f**) CMC/Ag hydrogel treatment, (g) CMC/HA/Ag hydrogel treatment. Objective × 40. Scale in μm.

**Figure 8:**
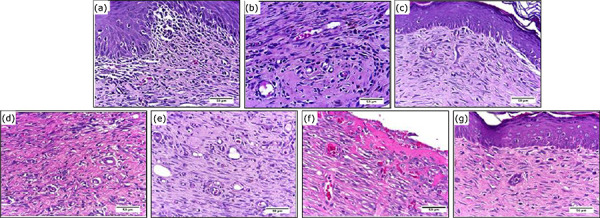
Photomicrograph of partial-thickness burn wounds experimentally induced in Wistar rats 30 days after the injury induction. (**a**) Physiologic solution (NaCl 0.9%) treatment, (**b**) 1% SS treatment, (**c**) 0.2% Hy, (**d**) CMC hydrogel treatment, (**e**) CMC/HA hydrogel treatment, (**f**) CMC/Ag hydrogel treatment, (**g**) CMC/HA/Ag hydrogel treatment. Objective × 40. Scale in μm.

The histopathologic analyses evaluated the intensity of the general pathologic processes during the inflammatory, proliferative and remodeling phases of the healing process, which are described in Table 2, and Figs. 6–8.

**Table 2:** Microscopic analysis of the general pathologic processes related to the healing process of partial-thickness burn wounds experimentally induced in Wistar rats.

Pathologic processes	DAI	Control (n = 18)			Hydrogels (n = 18)				p	Dunn’s
		PS	SS	Hy	CMC	CMC/HA	CMC/Ag	CMC/HA/Ag		
PMN infiltration	7	2 (1–3)	1 (1–2)	3 (3–3)	2 (1–3)	3 (2–3)	3 (3–3)	3 (3–3)	0.001	CMC/Ag > SS CMC/Ag > PS CMC/HA/Ag > SS CMC/HA/Ag > PS HY > SS HY > PS CMC/HA > SS
	14	1 (1–1)	2 (1–3)	1 (0–1)	1 (1–2)	1 (1–1)	1 (1–1)	2 (1–2)	0.005	SS > HY CMC/HA/Ag > Hy
	30	1 (1–0)	1 (0–1)	0 (0–0)	1 (1–2)	0 (0–0)	1 (0–2)	0 (0–1)	0.009	CMC > Hy CMC > CMC/HA CMC > PS
MN infiltration	7	2 (2–3)	3 (3–3)	2 (2–3)	2 (2–3)	2 (2–2)	3 (3–3)	2 (2–3)	0.002	SS > CMC/HA SS > CMC/HA/AG CMC/Ag > CMC/HA CMC/Ag > CMC/HA/Ag
	14	2 (2–3)	2 (2–2)	2 (1–2)	3 (2–30)	3 (2–3)	2 (2–3)	3 (2–3)	0.088	
	30	1 (1–2)	2 (1–2)	1 (1–1)	2 (1–2)	1 (0–1)	1 (1–2)	1 (1–2)	0.102	
Angiogenesis	7	3 (3–3)	3 (3–3)	3 (3–3)	3 (3–3)	3 (3–3)	3 (3–3)	3 (3–3)	1	
	14	3 (2–3)	2 (2–2)	3 (2–3)	3 (3–3)	3 (3–3)	3 (3–3)	3 (3–3)	0.001	CMC > SS CMC/HA > SS CMC/Ag > SS CMC/HA/Ag > SS PS > SS Hy > SS
	30	1 (1–1)	1 (1–2)	1 (1–1)	2 (1–2)	2 (2–2)	2 (1–3)	2 (1–3)	0.010	CMC/Ag > Hy CMC/Ag > PS

Results expressed in median (minimum – maximum). n: number of animals. DAI: days after the injury induction. PMN: polymorphonuclear cells; MN: mononuclear cells; PS: physiologic solution (NaCl 0.9%); SS: 1% silver sulfadiazine; Hy: 0.2% Hyaludermin; CMC: carboxymethyl chitosan; CMC/HA: carboxymethyl chitosan and hyaluronic acid; CMC/Ag: carboxymethyl chitosan and silver; CMC/HA/Ag: carboxymethyl chitosan, hyaluronic acid and silver. Statistical analysis: Kruskal Wallis and Dunn’s post-test.

In the inflammatory phase was possible to observe that the groups treated with CMC/Ag and CMC/HA/Ag presented greater intensity of polymorphonuclear (PMN) cells infiltration in comparison to the PS and SS control groups.

During the proliferative phase, the hydrogel treatments did not interfere in the general pathologic processes analyzed.

While in the remodeling phase, the groups treated with CMC presented greater intensity of PMN cells infiltration than the PS and Hy control groups. The CMC treated group also presented greater intensity of PMN cells infiltration than the CMC/HA treated group. Regarding angiogenesis, it was greater in the CMC/Ag treated group than the PS and Hy control groups.

**Figure 9:**
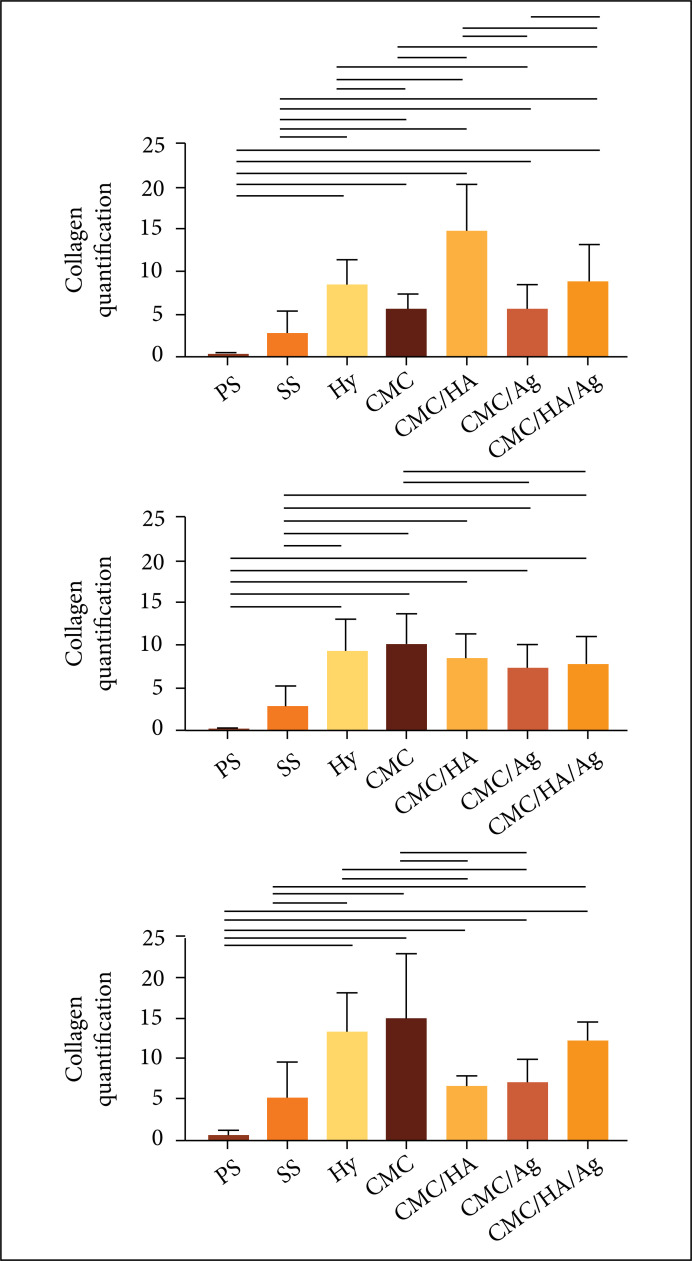
Microscopic evaluation of collagen fibers deposition in partial-thickness burn wounds experimentally induced in Wistar rats. Results expressed in mean ± standard deviation. DAI: days after the injury induction. PS: physiologic solution (NaCl 0.9%); SS: 1% silver sulfadiazine, Hy: 0.2% Hyaludermin, CMC: carboxymethyl chitosan, CMC/HA: carboxymethyl chitosan and hyaluronic acid, CMC/Ag: carboxymethyl chitosan and silver, CMC/HA/Ag:carboxymethyl chitosan, hyaluronic acid and silver. Horizontallines indicate the significant statistical difference. Statistical analysis: ANOVA and Tukey’s post-test.

Regarding the quantitative analysis of collagen fibers during the inflammatory phase, all the hydrogel treatments induced greater collagen quantification than the PS and SS control groups. When comparing the hydrogel treatments, the group treated with CMC/HA presented more collagen than the other treatments. The group treated with CMC/HA/Agpresented greater collagen than the CMC/Ag (Fig. 9).

In the proliferative phase, all the hydrogel treatment groups presented greater collagen quantification than the PSand SS control groups. The comparison between the treatments showed that the CMC treated group presented greater collagen deposition than the CMC/Ag and CMC/HA/Agtreated ones.

While in the remodeling phase, all the hydrogel treatments presented greater collagen deposition than the PS control group. The comparison between treatments showed that the CMC treated group presented more collagen deposition than CMC/HA and CMC/Ag treated ones.

## Discussion

Chitosan can be easily carboxymethylated to improve its solubility, biodegradability and biocompatibility. Carboxymethylation is an etherification reaction that consists of replacing the hydrogens present in the amino and hydroxyl groups of the chitosan structure,with carboxymethyl groups (CH2COOH)^29^,^35^. In the present study, carboxymethylation reaction of chitosan was carried out with monochloroacetic acid in isopropanol alcohol in alkaline conditions through a 24 h reaction. Sodium hydroxide reacts with chitosan and promotes the protonation of amino groups (-NH2) and/or hydroxyl groups (-OH) arranged in the molecule^36^, while monochloroacetic acid acts as a carboxymethylation agent, promoting reactions between chitosan groups to form CMC^37^.

After the carboxymethylation reaction carried out in this study, the comparison of the ^1^H NMR and FTIR spectra of chitosan and synthesized CMC confirmed that the chemical modifications of chitosan occurred successfully under the conditions used in this study. In the ^1^H NMR spectra of CMC, the presence of signs of the hydrogens of the carboxymethyl group linked to C (6) and C (3) was observed in 4.6 and 4.9 ppm, respectively, confirming data reported in the literature^29^,^31^. The signs of the carboxymethyl group hydrogens linked to the nitrogen of the amino group were observed in the chemical displacement interval 3.8 < δ < 4.0 ppm, with the occurrence of N-carboxymethylation (carboxymethylation in amino groups) in low extension/intensity^38^.

In the FTIR spectra of CMC, the main changes were observed in the range of 1621 and 1431 cm^–1^, attributed to the introduction of carboxylic groups in the polymer chain, similar to that described previously by other authors^19^,^29^,^39^. It has been reported that the carboxylic groups present in the CMC improve the hydrophilicity of the molecule, improving its use in the treatment of wounds^40^,^41^.

Scanning electron microscopy revealed that chitosan and CMC had a continuous flat surface with roughness under the polymer matrix, the lyophilized hydrogel of CMC and CMC/HA a well-developed porous structure and lyophilized hydrogel of CMC/Ag and CMC/AH/Ag microstructure with reliefs and fibrous appearance under the polymeric matrix. According to the literature, the porous surface of a dressing has great importance in the wound healing process, since it promotes a moist environment and oxygenation at the wound site and removes exudate^42^.

The TGA results showed that CMC and lyophilized hydrogels have a thermal degradation profile in three stages of mass loss. The first stage refers to the evaporation of water physically adsorbed in the polymeric network^25^ and started in the temperature range of 40 to 150 °C, with a mass loss of 3 to 14%. The second stage of mass loss, attributed to the beginning of thermal degradation of the pyranose ring of the polymeric chain^43^, started at about 290 °C and extends to 310 °C with a mass loss ranging from 30 to 40%. The third stage occurs at temperatures above 500 °C, corresponding to the decomposition of the carboxylic groups and glucosamine units of the polysaccharide structure^44^.

This study evaluated the healing process in partial-thickness burn wounds experimentally induced in Wistar rats after treatment with hydrogel formulations. The complex biological process of healing includes a wide range of macroscopic and microscopic parameters, such as necrosis/crust and granulation tissue formation, re-epithelialization, inflammatory cells infiltration, angiogenesis and collagen fibers deposition^45^. It has been shown that the composition of the dressing used on the care of different wounds are critical on the evolution of the healing process regarding several aspects, such as the ones evaluated in this study^46^.

The benefits of the isolated application of hydrogels, CMC and HA in wound dressings for the treatment of several types of wounds has been described previously^15^,^17^,^20^,^23^,^26^,^35^. However, the association of such substances and their beneficial effects on the healing process of burn wounds experimentally induced in rats is being described for the first time in this study.

The dressings evaluated in this study showed easy handling and appliance during the experiment period of 30 days and there was no sign of infection in the treated injuries. It is important to highlight that both chitosan and CMC favor the chemotaxis of neutrophils, which prevents the wound infection^38^,^47^–^50^. It has been demonstrated that CMC presents enhance biological properties when compared to chitosan, such as increased solubility and biocompatibility, greater viscosity and moisture retention, and improved antimicrobial activity. These enhancements demonstrate why CMC is a better choice than chitosan for a wound dressing material^35^.

During the inflammatory phase, the wound contraction was greater after the treatment with CMC hydrogel formulations. It was also possible to observe more granulation tissue, PMN inflammatory infiltration and collagen fibers deposition. The association of CMC with HA and Ag was the hydrogel formulation that mostly influenced these aspects at the initial days of the healing process.

Hydrogels are biomaterials with important healing properties, such as permeability to metabolites, nonirritant and nonreactive towards biological tissue. The high-water content of hydrogel formulations is important in the granulation tissue formation. Also, hydrogel has been considered suitable for all phases of the healing process^51^. Humidity has been described as an important factor regarding the collagen deposition, which is directly related to the contraction of the wound^52^.

On the other hand, chitosan has been shown to present an important effect as PMN chemotactic, inducing the production of complement 5a (C5a), induction of interleukin-8 (IL-8) and collagen production by *in situ* fibroblasts, therefore activating inflammatory migration into the wounded area inexperimental open wound in dogs^53^. These findings arein accordance to this study in which there is an increase in PMN inflammatory infiltration and collagen quantification after the chitosan hydrogels treatments.

Regarding the treatment of experimental burn wounds, chitosan has been shown to significantly induce a greater wound contraction and re-epithelialization of the injuries during the inflammatory phase, approximately up to 7 days after the injury induction^54^. Similar results to the ones found in this study. It has been reported important antimicrobial effect of chitosan alone and impregnated with Ag in the treatment of experimental burn wounds during the first days of the healing process^55^.

The addition of HA to the hydrogel formulation has been shown to contribute to the humidity of the environment, since it is highly osmotic and hygroscopic. It is an important stimulator of the cell proliferation and migration^56^. These findings explain why the groups that presented HA in addition to CMC presented higher PMN inflammatory infiltration, granulation tissue and collagen deposition.

During the proliferative phase of the healing process, the hydrogel treatments induced greater wound contraction and collagen fibers deposition. It was also possible to observe less necrosis after the hydrogel treatments. The association of CMC with HA was the formulation that better influenced these aspects.

The addition of HA to the CMC formulation increased its effect on the healing process. It stimulates the proliferation and migration of epithelial cells, modulates inflammatory responses, increases angiogenesis and eliminates oxygen reactive species derived from inflammatory cells. It also increases the collagen fibers deposition in the wound site^13^,^57^–^61^.

The efficacy of the association of chitosan, HA and arginine in promoting the healing process in experimental burn wounds in rats have been described previously^14^,^18^. As well as its different formulations, such as dextran-based hydrogels loaded with chitosan microparticles containing growth factor induced fastest healing burn wounds experimentally induced in rats^33^.

While, during the remodeling phase, the hydrogel treatments induced greater wound contraction, re-epithelialization, angiogenesis and collagen fibers deposition with less necrosis. As observed in the proliferative phase, the association of CMC with HA was the formulation with best influence on these aspects.

Polymeric membranes containing chitosan, HA and arginine derivates have shown healing properties regarding experimentally induced burn wounds in rats, demonstrating the potential of such polymers as burn wound dressings. The use of such dressings increased the wound contraction^14^, which is in accordance with the results of this research. Chitosan and HA have been shown to induce greater wound closure also in excisional wounds experimentally induced in rats, improving the healing process^25^.

The CMC associated to HA treatments induced lower necrosis/crust formation than the traditional Ag-based treatment during the inflammatory, proliferative and remodeling phases of the healing process. Also, the granulation tissue formation and the re-epithelialization were induced by the CMC/HA treatment. As described previously, the appliance of a N-carboxymethyl chitosan membrane has significantly improved the healing rate of burn wounds experimentally induced in rats regarding the reduction in necrosis, increase in granulation tissue and re-epithelialization^62^. These results may be explained by the improved humidity, cytocompatibility and antibacterial activity of chitosan^63^,^64^. The enhanced re-epithelialization has been described in natural polymers alongside with the characteristics of hemostatic, antibacterial, anti-inflammatory and wound healing properties^12^,^58^.

The increase in angiogenesis observed in the hydrogel treated groups during the remodeling phase is explained by the induction of vascular endothelial growth factor expression, as described in the study of the effect of carboxymethylcellulose sodium/sodium alginate/chitosan hydrogel in deep second-degree burn wounds^64^. Other important effects observed were the down regulation of basic fibroblast growth factor in the early periods of wound healing and up regulation at a later stage and decreased levels of tumor necrosis factor alpha (TNF-alpha) and interleukin-6 (IL-6)^64^.

It has been demonstrated previously that N-carboxymethyl chitosan formulations induced reduction in wound size, accelerated wound healing and greater collagen deposition in deep second-degree burn wounds experimentally induced in rats^62^, which is in accordance to the collagen quantification in these results. *In vitro* studies have demonstrated the chitosan capability of stimulating fibroblasts and keratinocytes, which are important cells in the healing process, especially in the collagen production^11^,^49^,^61^.

## Conclusions

The carboxymethylation reaction was carried out successfully and was efficient in the hydrogel production. The hydrogels consisting of CMC, HA and Ag nitrate in different formulations indicated good compatibility between the components and thermal stability. This study is the first description of the effect of CMC associated to HA and Ag on the healing process of partial-thickness burn wounds experimentally induced in rats. The CMC hydrogel treatments showed positive effects in some aspects of the healing process. It was possible to determine that, in the early phases of the healing process, during the inflammatory phase, the use of the association of CMC with HA and Ag is the best formulation in order to enhance several aspects of the healing process. While in the following phases of the healing process, proliferative and remodeling ones, the best formulation in order to enhance the healing process is CMC associated to HA.
